# Development and characterization of a human Th17‐driven ex vivo skin inflammation model

**DOI:** 10.1111/exd.14160

**Published:** 2020-08-25

**Authors:** Claire Jardet, Anthony David, Emilie Braun, Pascal Descargues, Jean‐Louis Grolleau, Josephine Hebsgaard, Hanne Norsgaard, Paola Lovato

**Affiliations:** ^1^ Genoskin Toulouse France; ^2^ Genoskin Inc. Salem Massachussetts USA; ^3^ Service de Chirurgie Plastique et Reconstructrice CHU Toulouse Rangueil Toulouse France; ^4^ Skin Research LEO Pharma A/S Ballerup Denmark

**Keywords:** epidermal activation, IL‐17 immune axis, IL‐23, skin‐resident T‐cell activation, topical treatment

## Abstract

Skin models mimicking features of psoriasis‐related inflammation are needed to support the development of new drugs in dermatology. Reconstructed skin models lack tissue complexity, including a fully competent skin barrier, and presence and/or diversity of immune cells. Here, we describe InflammaSkin®, a novel human Th17‐driven ex vivo skin inflammation model. In this model, skin‐resident T cells are in situ activated by intradermal injection of anti‐CD3 and anti‐CD28 antibodies and Th17 cell polarization is sustained by culture in a chemically defined medium supplemented with IL‐1β, IL‐23 and TGF‐β for seven days. The acquired Th17 signature is demonstrated by the sustained secretion of IL‐17A, IL‐17AF, IL‐17F, IL‐22, IFN‐γ, and to some degree IL‐15 and TNF‐α observed in the activated ex vivo skin inflammation model compared with the non‐activated skin model control. Furthermore, expression of S100A7 and Keratin‐16 by keratinocytes and loss of epidermal structure integrity occur subsequently to in situ Th17cell activation, demonstrating cellular crosstalk between Th17 cells and keratinocytes. Finally, we demonstrate the use of this model to investigate the modulation of the IL‐23/IL‐17 immune axis by topically applied anti‐inflammatory compounds. Taken together, we show that by in situ activation of skin‐resident Th17 cells, the InflammaSkin® model reproduces aspects of inflammatory responses observed in psoriatic lesions and could be used as a translational tool to assess efficacy of test compounds.

## 1 INTRODUCTION

The IL‐23/IL‐17 immune axis is of key importance for driving skin inflammation in psoriasis, which results from the interplay between keratinocytes and immune cells, such as Th17 cells. Pharmacological blockade of this immune axis by biologics has shown impressive clinical efficacy in psoriasis. In order to study pharmacological modulation of this immune axis by different drug modalities and administration (topical and systemic), there is a need for a relevant human skin model that includes cellular crosstalk between Th17 cells and keratinocytes. Human Th17 differentiation requires IL‐1β, IL‐6 and TGF‐β as well as IL‐23 to sustain production of IL‐17 and IL‐22 from Th17 cells. IL‐17 induces expression of other pro‐inflammatory mediators in keratinocytes, such as IL‐17C, IL‐19 and IL‐36 that, together with IL‐17 and IL‐22, contribute to keratinocyte activation and epidermal hyperplasia, associated with expression of Keratin‐16 and S100A7.^[^ ^1^ ^]^ In addition, increased levels of IFN‐γ in psoriatic skin by activation of skin‐resident Th1 cells contribute to the inflammatory activation of keratinocytes.^[^ ^1^ ^]^

Several skin models have been published which mimic some aspects of psoriasis‐related inflammation, using keratinocyte cultures, reconstructed skin models (derived from healthy or psoriatic tissue), or skin explants, which are stimulated with cytokines to induce inflammatory responses in keratinocytes.^[^ ^2^, ^3^, ^4^, ^5^, ^6^, ^7^, ^8^, ^9^ ^]^ Such models exclude investigation of cellular interplay as activated immune cells/Th17 cells are not present. In a publication by van den Bogaard et al,^[^ ^10^ ^]^ a human reconstructed skin model with incorporation of polarized CD4^+^ T cells was described allowing crosstalk between keratinocytes and T cells.^[^ ^10^ ^]^ Reconstructed skin models, although containing a stratified epidermis, lack a fully competent skin barrier and the exact cellular architecture and compartments, such as the different immune cell types, which are all present in human skin, imposing limitations for pharmacological studies of topically applied drugs or therapies targeting specific immune cell types in psoriasis.

Here, we describe a novel human Th17‐driven skin inflammation model (InflammaSkin®) based on in situ activation of skin‐resident Th17 cells in the NativeSkin® model, a full thickness ex vivo skin model that retains physiological skin biology, cell viability, skin barrier function and metabolism over 7 days of culture.^[^ ^11^ ^]^ This skin model includes crosstalk between Th17 cells and keratinocytes and reproduces aspects of the inflammatory responses observed in psoriatic lesions. Furthermore, we demonstrate the use of this model to investigate modulation of the IL‐23/IL‐17 immune axis by topically applied anti‐inflammatory compounds.

## 2 METHODS

### 2.1 Test reagents

0.5 mg/g betamethasone dipropionate gel and 2.5 mg/g LEO 29102 (PDE4 inhibitor) cream with corresponding placebo formulations were manufactured by LEO Pharma.

### 2.2 Production and culture of the InflammaSkin® model

Anonymized human skin samples were obtained from donors that underwent abdominoplasty procedures and had given their written informed consent. Donors did not have any record of allergies or dermatological disorders and did not use corticosteroids. Full ethical approval for the study protocol was obtained from the French ethical research committee (Comité de Protection des Personnes) and authorization was given from the French ministry of Research. All studies were conducted according to the Declaration of Helsinki protocols. Performed studies were conducted on female Caucasian donors aged between 32 and 64.

Immediately following surgery, skin samples were transported at room temperature before being processed. Subcutaneous adipose tissue was removed from the skin sample. 8‐mm‐diameter punch biopsies were excised and intradermally injected with an activation cocktail containing 10 ng/mL recombinant human (rh)IL‐2 (202‐IL, Bio‐Techne), 50 µg/mL free anti‐CD3 antibody and 50 µg/mL free anti‐CD28 antibody (130‐091‐441, Miltenyi Biotec) in order to activate skin‐resident T cells. Biopsies were embedded in a proprietary biological matrix in transwells (Millicell) according to the patented NativeSkin® procedure developed by Genoskin. The epidermal surface of skin biopsies was left in contact with the air, and the dermal compartment was immersed in the matrix. The skin models were cultured up to 7 days in 12‐well plates in a proprietary and chemically defined hydrocortisone‐ and serum‐free medium in the presence of 100 µg/mL penicillin and 100 µg/mL streptomycin, and supplemented with a Th17 polarization cocktail containing 10 ng/mL rhIL‐1β protein (201‐LB, Bio‐Techne), 50 ng/mL rhIL‐23 protein (1290‐IL, Bio‐Techne) and 10 ng/mL rhTGF‐β protein (240‐B, Bio‐Techne) in a humidified atmosphere of 5% CO_2_ at 37°C.

For treatment studies, 10 µL of each formulation was topically applied once daily either from the start of in situ activation (prophylactic setting; from day 0 to day 6 of culture) or after inflammation was induced (therapeutic setting; from day 4 ‐ day 6 of culture).

### 2.3 Sampling

The culture supernatants were collected and snap frozen at −80°C for cytokine analysis. Skin biopsies were split into two parts; one part was fixed in 10% buffered formalin solution (HT501128‐4, Sigma‐Aldrich) at room temperature and processed for paraffin wax embedding. 5‐µm‐thick skin cross‐sections were prepared using a microtome for histological analysis. The second part was snap frozen at −80°C for gene expression analysis.

### 2.4 Histological and immunofluorescence analyses

Haematoxylin and eosin (10 047 105, VWR and HT110116, Sigma‐Aldrich) staining was performed to assess skin structure. Transmitted‐light images were acquired with an optical microscope (Leica DMi1) and a Leica MC170HD camera connected to a computer with Leica Application Suite (LAS®) for image capture.

Anti‐Keratin‐16 (SAB4501660, Sigma‐Aldrich) and anti‐S100A7 (HPA006997, Sigma‐Aldrich) immunofluorescence stainings were performed to evaluate psoriasis‐related epidermal hyper‐proliferation, activation and skin barrier integrity, respectively. Anti‐Ki67 (M724029, Dako) and anti‐active Caspase‐3 (ab2302, Abcam) immunofluorescence stainings were performed to evaluate proliferation and apoptosis in the model, respectively. Anti‐Loricrin (PRB‐145P‐100, Covance), anti‐Involucrin (J64013, CliniSciences) and anti‐(pro)‐Filaggrin (AHF3 clone) immunofluorescence stainings were performed to evaluate changes in epidermal terminal differentiation. Cross‐sections were stored at 60°C for 1 hour prior to antigen retrieval step, saturation and incubation with antibodies for overnight at 37°C. A specific signal was detected using secondary antibodies conjugated to Alexa Fluor 555 or Alexa 647 dye (Invitrogen Life Technologies). DAPI (D9542, Sigma‐Aldrich) was used to counterstain skin sections for the immunofluorescence analyses. Images were obtained with a widefield microscope (Leica DM5000B) and a CoolSNAP EZ CCD camera (Photometrics) controlled with the MetaVue software (Molecular devices) with strictly the same parameters and duration of exposure. Images were processed with ImageJ software.

### 2.5 Quantitative real‐time (qRT‐PCR) analysis

Total RNA was extracted by using the mirVana kit (Life Technologies) according to the instructions provided by the manufacturer and Precellys® 24 tissue homogenizer (Bertin instruments) for the mechanical lysing of the tissue. cDNA synthesis was performed with the High‐Capacity cDNA Reverse Transcription Kit (Applied Biosystems). cDNA was amplified by quantitative real‐time PCR using the following Taqman® Gene Expression Assays (IL4‐Hs00174122_m1, IL10‐Hs00961622_m1, IL13‐Hs99999038_m1, IL15‐Hs00542571m1, IL‐17A‐Hs00174383_m1, IL‐17F‐Hs00369400_m1, IL22‐Hs01574154_m1, IFNg‐Hs00989291_m1, TNFa‐Hs01113624_g1, ICAM1‐Hs00164932_m1, FLG‐Hs00856927_g1, PPIA‐Hs99999904_m1, GAPDH‐Hs99999905_m1, ACTB‐Hs01060665_g1, RPLP0‐Hs99999902_m1). PPIA, ACTB and RPLPO were used as reference genes in the model characterization studies, whereas PPIA and GAPDH were used as reference genes in the pharmacological treatment studies.

### 2.6 Cytokine analysis

Levels of cytokines secreted in the culture supernatants were quantified using Meso Scale Diagnostic (MSD) kits for IL‐4, IL‐10, IL‐13, IL‐15, IL‐17A, IFN‐γ, TNF‐α (U‐plex multiplex kit), for IL‐17AF (U‐plex as single kit) and ELISA kit for IL‐22 (R&D Systems) following manufacturer's instructions. Cytokine concentrations expressed in pg/mL were calculated by comparison to the standard curve, also expressed in pg/mL, which was generated in the same biological matrix as the samples. The lowest limit of detection (LLOD) was 0.1, 0.2, 4.1, 1.4, 3.67, 10, 0.4‐3.8, 2.3‐2.9, 0.1‐0.3 and 10‐25 pg/mL for IL‐4, IL‐10, IL‐13, IL‐15 IL‐17AF, IL‐17F, IL‐17A, IFN‐γ, TNF‐α and IL‐22, respectively.

### 2.7 Statistical analysis

Statistical analysis was performed using the GraphPad Prism 8.1.1 software.

For model characterization studies, statistical analysis was performed by two‐way analysis of variance (ANOVA) followed by Tukey's multiple comparison tests for both gene expression and protein analysis. Values are shown as mean ± SEM of delta‐Ct values (ΔCt) and of pg/mL, respectively.

For analysis of cytokine secretion in the pharmacological studies, results were expressed as percentage of untreated controls for each experiment, and statistical analysis of treatment effects was performed by one‐way analysis of variance (ANOVA) followed by Tukey's multiple comparison tests.

## 3 RESULTS

### 3.1 Characterization of the InflammaSkin® model

**FIGURE 1:**
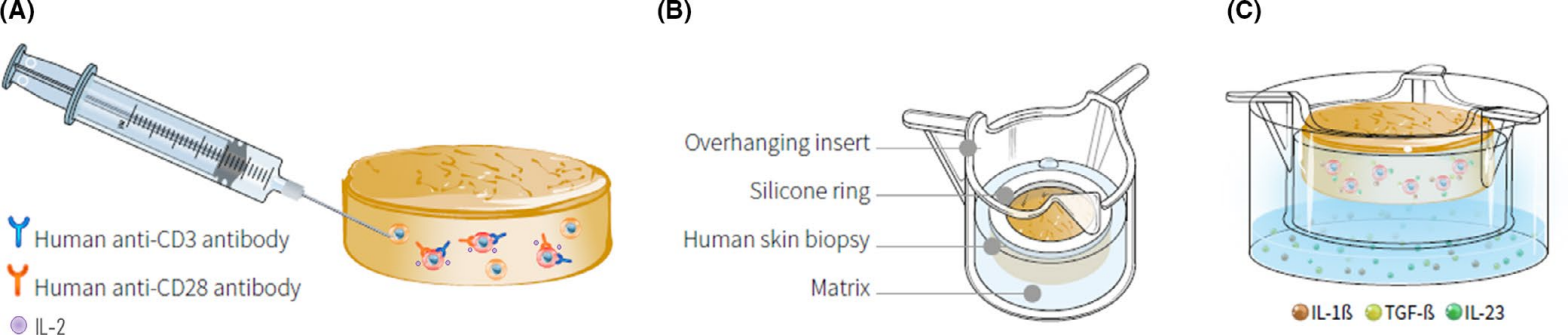
Schematic representation of InflammaSkin® model production. InflammaSkin® models were created from NativeSkin® technology.Biopsies were injected with rhIL‐2, anti‐human CD3 and anti‐human CD28 antibodies to activate skin‐resident T cells (A). Injected biopsies were then embedded in a proprietary biological matrix in transwells (B). InflammaSkin® models were cultured at a humidified atmosphere with 5% CO2 at 37°C in a chemically defined culture medium supplemented with a cocktail containing recombinant human IL‐1β, TGF‐β and IL‐23 to sustain a Th17 phenotype (C)

In order to develop a human ex vivo T cell‐driven skin inflammation model that reproduces inflammatory responses associated with psoriasis, our approach relied on activation of skin‐resident Th17 cells present in normal skin, using NativeSkin® skin explants. The InflammaSkin® model was produced in three different steps (Figure 1). Activation of resident T cells in the skin biopsies was induced by intradermal injection of an activation cocktail containing rhIL‐2 and anti‐CD3 and anti‐CD28 antibodies (Figure 1A). Skin biopsies were then embedded into the patented NativeSkin® proprietary matrix and culture system that has been previously demonstrated to enable maintenance of normal skin viability and histological features for 7 days of ex vivo culture (Figure 1B). Finally, the NativeSkin® chemically defined and serum‐free culture medium was supplemented with a polarization cocktail containing pro‐inflammatory cytokines IL‐1β, IL‐23 and TGF‐β in order to sustain the Th17 phenotype (Figure 1C).

**FIGURE 2:**
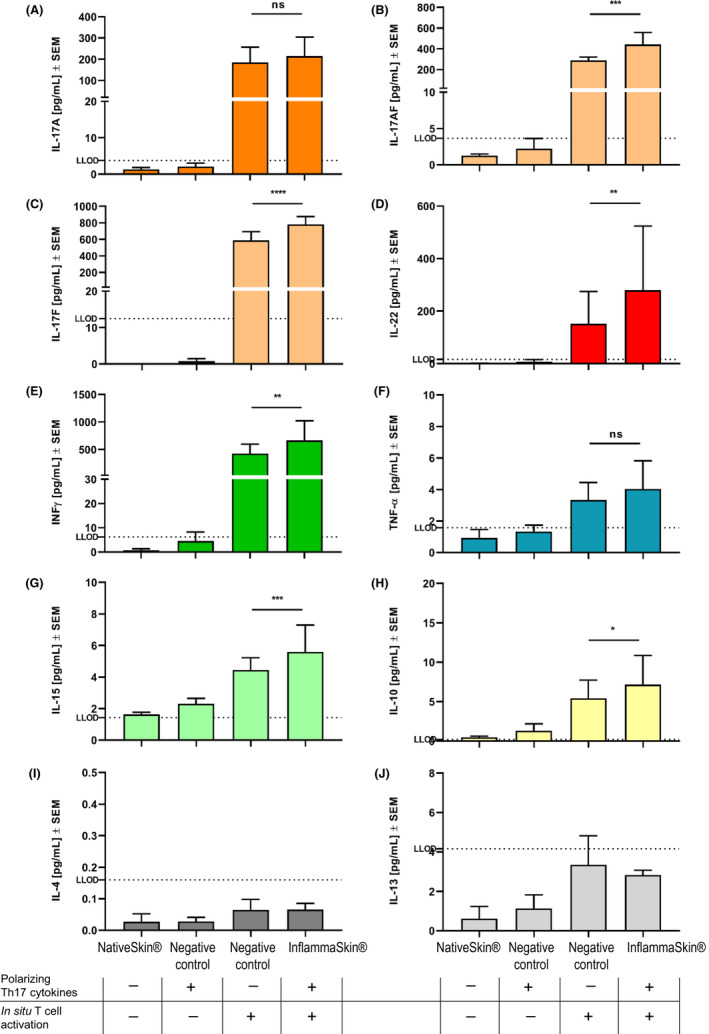
Characterization of cytokine release consecutive to Th17/Th1 activation. NativeSkin® models were either cultured under basal conditions or in the presence of the Th17 polarization cocktail (negative control), or injected with rhIL‐2, anti‐CD3/CD28 antibodies (in situ activation) in the absence (negative control) or presence of the Th17 polarization cocktail (InflammaSkin®) for 7 days. Protein levels of IL‐17A (A), IL‐17AF (B), IL‐17F (C), IL‐22 (D), IFN‐γ (E), TNF‐α (F), IL‐15 (G), IL‐10 (H), IL‐4 (I) and IL‐13 (J) were measured by ELISA (IL‐22) and MSD (all the other cytokines) in supernatant samples harvested after the last 24 h of 7 d of culture. Lowest limit of detection (LLOD) for each cytokine is indicated by a dotted line. Data are expressed as mean values ± SEM of pg/mL of secreted cytokines of models obtained from three different skin donors (n = 3). Two‐way ANOVA statistical analysis followed by Tukey's multiple comparison tests was used to compare protein levels. ****P < .0001; ***P < .001; **P < .01; *P < .05; ns = not significant

A time course study was performed to assess the optimal window for the induction of T cell–mediated inflammation. Therefore, T cell–activated models were cultured in the absence or presence of the polarization cocktail for either 3, 5 or 7 days. Expression of the Th17 cytokines IL‐17A and IL‐22 was induced at day 3 with highest expression at day 7 (Figure S1A). However, expression of IFN‐γ was also induced at day 3 but peaked at day 5 (Figure S1A), indicating that activation of Th1 cells also occurs following in situ activation despite the presence of the Th17 polarization cocktail. No secretion of these T‐cell cytokines were seen in the non‐activated controls with or without the polarization cocktail (Figure S1A). The Th2 cytokine IL‐4 was also tested and was not detectable in any of the treatment conditions (data not shown). Analysis of skin morphology by haematoxylin and eosin (H&E) staining revealed a negligible degeneration in the epidermal crest in response to the Th17 polarizing cocktail only (Negative control, Figure S1B, panel b). This epidermal change was observed from day 3 (data not shown). Furthermore, in the InflammaSkin® model the H&E staining revealed the appearance of first signs of cell degeneration with however no loss of viability as soon as day 3 of culture, characterized by spongiosis and cell vacuolization in all layers of the epidermis (Figure S1B, panel c). From day 5 up to day 7, numerous pyknotic nuclei as well as epidermal loss of integrity and detachment were observed, suggesting an important loss of cell viability (Figure S1B, panels d and e). Based on the time course study, the Th17/Th1 phenotype was evaluated in different skin donors by the level of T cell‐related cytokines secreted in the culture medium upon in situ T‐cell activation and culture with the Th17 polarization cocktail for 7 days (secreted cytokines measured from the last 24 hrs of culture). NativeSkin® models were cultured as control conditions in the absence or presence of the polarization cocktail (negative control without activation) or the T‐cell activation only (negative control without Th17 polarization cocktail). The study was carried out in three independent experiments containing a total of three donors. Gene expression analysis of skin biopsies (Figure S2) and protein analysis of supernatants (Figure 2) showed pronounced expression of IL‐17 family cytokines (IL‐17A, IL‐17AF, IL‐17F), IL‐22, IFN‐γ and to some degree IL‐15 and TNF‐α in response to injection of IL‐2 and anti‐CD3/anti‐CD28 antibodies, indicating that in situ T‐cell activation was sufficient to activate both Th17 and Th1 cells. Importantly, the presence of the Th17 polarization cocktail (*eg* IL‐1β, IL‐23 and TGF‐β) allowed a more pronounced expression of all pro‐inflammatory cytokines related to Th17 and Th1 cells as shown by a significantly higher expression in the InflammaSkin® model compared with skin models cultured in the absence of the Th17 polarizing cocktail after in situ T‐cell activation (Figure 2 and Figure S2).

The Th2‐related cytokines, IL‐4 and IL‐13, were not detected by either gene expression or protein analysis (Figure S2 and Figure 2), indicating that Th2‐cell activity was not present in any of the tested conditions. Expression of ICAM‐1 was also assessed by gene expression and found to be upregulated upon in situ activation of T cells (Figure S2), supporting a pro‐inflammatory phenotype of activated skin T cells. Finally, the expression of IL‐10, assessed by both gene expression and protein analysis, was found to be induced by in situ activation of T cells and significantly higher expression was noted in the InflammaSkin® model compared to skin models cultured upon in situ T‐cell activation only (Figure S2 and Figure 2), indicating that regulatory pathways were also induced in skin models upon T‐cell activation.

**FIGURE 3:**
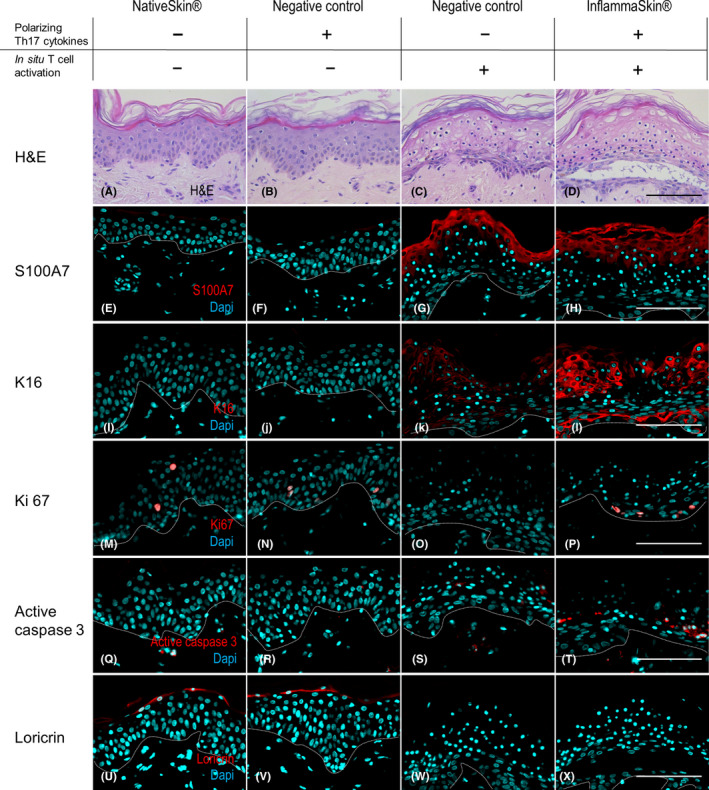
Characterization of histological changes consecutive to induction of Th17/Th1 inflammation. NativeSkin® models were either cultured under basal conditions or in the presence of the Th17 polarization cocktail (negative control), or injected with rhIL‐2, anti‐CD3/CD28 antibodies (in situ activation) in the absence (negative control) or presence of the Th17 polarization cocktail (InflammaSkin®) for 7 days. Haematoxylin and eosin staining (A‐D) was performed on 5‐µm‐thick skin cross‐sections. All images are representative of the whole sample. Scale bar is 100 µm. Anti‐S100A7 (E‐H), anti‐Keratin 16 (K16) (I‐L), anti‐Ki67 (M‐P), anti‐active Caspase‐3 (Q‐T) and anti‐Loricrin (U‐X) immunofluorescence stainings were performed on 5‐µm‐thick skin cross‐sections. Red and cyan signals indicate positive signal and DAPI‐counterstained nuclei, respectively. Dotted white lines represent the epidermal‐dermal junction. Epidermal and dermal compartments are localized above and below the dotted line, respectively. All images are representative of the whole sample. Scale bars are 100 µm

In order to assess epidermal responses consecutive to the activation of Th17 cells, skin structure integrity and expression of epidermal markers were investigated by immunofluorescent staining. Representative results of one donor from three independent experiments with a total of three donors are shown in Figure 3 and Figure S3. After 7 days of culture, haematoxylin and eosin (H&E) staining showed epidermis detachment, presence of numerous pyknotic nuclei and cellular vacuolization, indicating loss of viability and structure integrity of the epidermis in the InflammaSkin® model (Figure 3, panel D). Similar features were noted in the activated control models cultured in the absence of the polarization cocktail (Figure 3, panel C). Non‐activated controls cultured in the absence of the polarization cocktail (standard NativeSkin® model) showed normal epidermal structure and viability, and in the presence of the polarization cocktail, controls displayed only slight signs of degeneration with no loss of viability, characterized by mild spongiosis and a few vacuolated cells (Figure 3, panels A and B).

Expression of S100A7 and Keratin‐16, two epidermal markers upregulated in psoriasis lesions, was strongly induced in the suprabasal layers of the epidermis in the InflammaSkin® model (Figure 3, panel H and L), suggesting the presence of Th17‐mediated epidermal inflammation. Notably, expression of S100A7, but only low expression of Keratin‐16, was detected upon T‐cell activation alone (Figure 3, panel G and K), whereas no expression was found in the absence of T‐cell activation (Figure 3, panel E, F, I, J). The proliferation marker Ki67 was decreased in the two negative controls compared with the NativeSkin® control (Figure 3, panel N and O vs panel M), whereas a slight increase in Ki67‐positive cells was found in the InflammaSkin® model (Figure 3, panel P). Active Caspase‐3, a marker of apoptosis, was induced in the InflammaSkin® model but not in any of the control models (Figure 3, panel Q‐T).

Finally, expression of epidermal differentiation markers was assessed. Loricrin was absent in the InflammaSkin® model as well as in the T cell–activated skin model but present in the non‐activated control models (Figure 3, panel U‐X). Involucrin was found in the suprabasal layers of the epidermis in the InflammaSkin® model as well as in the activated control cultured in the absence of the polarization cocktail (Figure S3, panels c and d), whereas it was found in the granular layer of the epidermis in the NativeSkin® model and in the non‐activated negative control (Figure S3, panels a and b).

Expression of (pro)‐Filaggrin did not seem to be dysregulated neither in the InflammaSkin® model nor in the negative controls (Figure S3, panel f, g and h) compared with the NativeSkin® model (Figure S3, panel e).

### 3.2 Response of the InflammaSkin® model to topically applied anti‐inflammatory drugs

We next investigated the ability of the InflammaSkin® model to respond to topically applied anti‐inflammatory drugs of therapeutic relevance for psoriasis. Two treatments were used in this study: a gel containing 0.5 mg/g betamethasone dipropionate (BDP) and a cream containing 2.5 mg/g LEO 29102, a PDE4 inhibitor which showed efficacy in a four week psoriasis plaque study (NCT00875277). Corresponding placebo gel and cream were used as controls for betamethasone gel and PDE4 inhibitor cream treatments, respectively. Untreated InflammaSkin® model was used as a positive control. Formulations were topically applied once daily either from the start (prophylactic setting; from day 0 to day 6 of culture) or after inflammation was induced (therapeutic setting; from day 4 to day 6 of culture). The therapeutic setting was based on the results from the time course study. The study was conducted on three donors (independent experiments).

**FIGURE 4:**
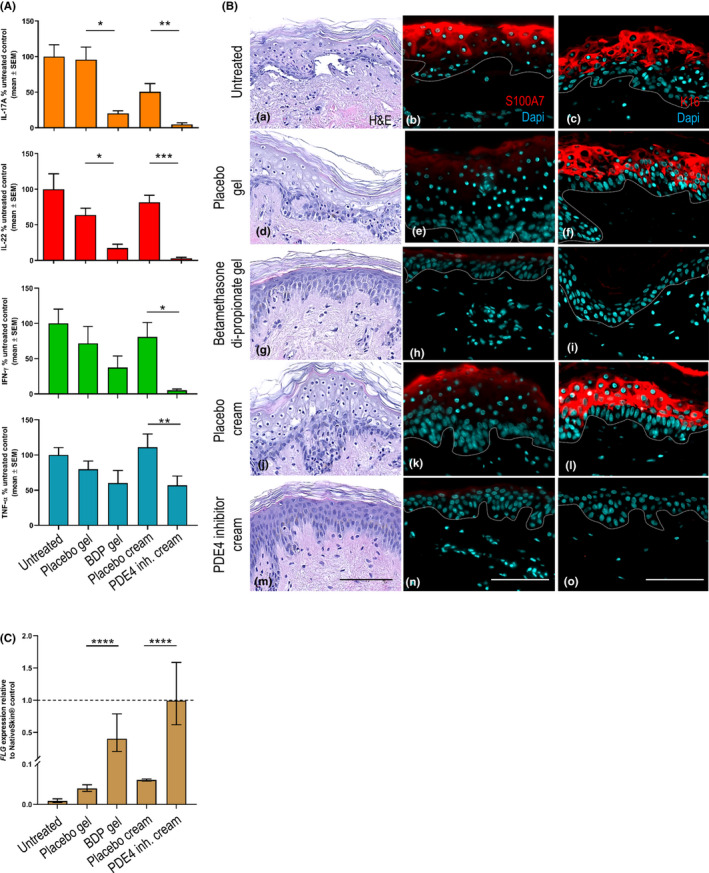
Pharmacological response of the InflammaSkin® model to prophylactic treatment with BDP gel and PDE4 inhibitor cream. InflammaSkin® models were cultured for 7 d either untreated or topically treated with placebo gel, Betamethasone dipropionate gel (BDP gel), placebo cream or PDE4 inhibitor cream (PDE4 inh. cream), applied every day from day 0 to day 6. At Day 7, skin models were fixed in 10% buffered formalin solution and processed for paraffin wax embedding prior specific histological staining and culture media were assessed for the presence of Th17/Th1‐related inflammatory cytokines. A, Levels of cytokines released in the culture media were measured using specific MSD for IL‐17A (orange graph), IFN‐γ (green graph) and TNF‐α (blue graph) and ELISA for IL‐22 (red graph). The results expressed as percentage difference compared with untreated control are mean values ± SEM from three independent experiments (n = 3). One‐way ANOVA statistical test was used to compare treatment effect by BDP and PDE4 inhibitor versus matching vehicle control. ***P < .001; **P < .01; *P < .05. B, Haematoxylin and eosin staining (panels a, d, g, j and m) was performed on 5‐µm‐thick skin cross‐sections. All images are representative of the whole sample. Scale bar is 100 µm. Anti‐S100A7 (panels b, e, h, k and n) and anti‐K16 (panels c, f, i, l and o) immunofluorescence staining were performed on 5‐µm‐thick skin cross‐sections. Red and cyan signals indicate positive signal and DAPI‐counterstained nuclei, respectively. Dotted white lines represent the epidermal‐dermal junction. Epidermal and dermal compartments are localized above and below the dotted line, respectively. All images are representative of the whole sample. Scale bars are 100 µm. C, *FLG* gene expression was assessed by real‐time quantitative PCR. The results are expressed as fold change relative to the NativeSkin® control ± upper and lower limits (n = 3). Two‐way ANOVA statistical test was used to compare treatment effect by BDP and PDE4 inhibitor versus matching vehicle control. ^****^ *P* < .0001

Compared with the untreated InflammaSkin® model and placebo controls, the levels of IL‐17A, IL‐22 and IFN‐γ were significantly inhibited by treatment with the PDE4 inhibitor applied in the prophylactic setting (Figure 4A). Comparable results were observed upon treatment with BDP in a similar setting, although the inhibition of IFN‐γ was not significant compared with its matching vehicle control. Both treatments resulted in a weaker effect on TNF‐α expression. The placebo cream also inhibited the expression of IL‐17A to some extent, but not of IL‐22, IFN‐γ and TNF‐α, while the placebo gel did not have any significant effect on the cytokines analysed.

The untreated InflammaSkin® model displayed loss of structure integrity and cell viability (Figure 4B, panel a) and showed strong expression of S100A7 and Keratin‐16 in the epidermis (Figure 4B, panels b and c), similar to previously obtained results. Application of placebo formulations, both gel and cream, showed similar profiles as the untreated InflammaSkin® model (Figure 4B, panels d to f, and j to l), except for S100A7 expression that seemed to be decreased after treatment with the placebo gel (Figure 4B, E). Interestingly, treatment with the BDP gel resulted in normal epidermal structure and viability apart from a mild spongiosis observed on H&E staining (Figure 4B, panels a and g), as well as a strong decrease in S100A7 expression, with only very low signal detected in the upper layer of the epidermis (Figure 4B, panels b and h), and no expression of Keratin‐16 (Figure 4B, panels c and i). Similar results were observed following treatment with the PDE4 inhibitor cream, which led to full preservation of skin structure and viability (Figure 4B, panel m), and complete decrease of both S100A7 and Keratin‐16 expression (Figure 4B, panels n and o).

To assess the impact on markers of the skin barrier, expression of filaggrin (*FLG*) was analysed by quantitative real‐time PCR. Compared with the NativeSkin® control, expression of *FLG* was almost completed inhibited in the untreated InflammaSkin® model (Figure 4C). Application of both placebo formulations resulted in a minor, statistically significant, upregulation of *FLG* expression versus the untreated InflammaSkin® model. Interestingly, *FLG* expression was partially restored by treatment with the BDP gel and fully restored by the PDE4 inhibitor cream, both statistically significant against the untreated InflammaSkin® model (Figure 4C).

Application of BDP and the PDE4 inhibitor after inflammation was induced (therapeutic setting) also inhibited the expression of IL‐17A, IL‐22 and IFN‐γ compared with the untreated InflammaSkin® model, however to a lesser extent compared with the prophylactic treatment (Figure S4 compared with Figure 4A). The placebo gel did not affect the expression of the cytokines whereas the placebo cream led to decreased expression of IL‐17A, IL‐22 and IFN‐γ comparable to the effect by the PDE4 inhibitor cream. TNF‐α expression was inhibited by the PDE4 inhibitor, but not by BDP (Figure S4). In this setting, the treatments were not able to restore normal skin structure and viability nor inhibit S100A7 and Keratin‐16 protein expression by immunofluorescent staining likely due to the short treatment time. However, *FLG* gene expression was partially restored by treatment with the BDP gel but not by the PDE4 inhibitor cream (Figures S5 and S6).

## 4 DISCUSSION

There are several human in vitro models addressing different aspects of psoriasis‐related inflammation. However, to the best of our knowledge, most of these models lack tissue complexity, complete skin barrier function and/or diversity of skin‐resident immune cells, since they are based on either keratinocyte cultures, reconstructed skin models or skin explants stimulated with cytokines to induce inflammatory responses in keratinocytes without activation of skin immune cells.^[^ ^2^, ^3^, ^4^, ^5^, ^6^, ^7^, ^8^, ^9^ ^]^ Therefore, there is a need for translational models that capture the interplay between activated resident T cells and keratinocytes to support pharmacological testing of novel anti‐inflammatory drugs. Thus, the aim of our work was to develop a Th17‐driven skin inflammation model (InflammaSkin®) where activated skin‐resident Th17 cells promote inflammatory responses in keratinocytes mimicking features of skin inflammation observed in vivo in psoriasis skin lesions.

A prerequisite for our model has been a successful in situ activation of skin‐resident Th17 cells. T‐cell activation and differentiation rely on specific signals from the T‐cell receptor and costimulatory receptors, predominantly CD28, and from specific differentiation pathways activated by additional environmental signals, predominantly cytokines. This tightly regulated step‐wise process leads to the generation of diverse phenotypic and functional T‐cell populations, which are characterized by the capacity to secrete phenotype‐related cytokines and effector molecules to exercise their cellular function.^[^ ^12^ ^]^ In particular, Th1 differentiation requires IL‐12 and IFN‐γ to sustain IFN‐γ production by Th1 cells, while Th17 differentiation requires the concomitant stimulation by IL‐1β, IL‐6, TGF‐β and IL‐23 to sustain production of IL‐17 and IL‐22 from Th17 cells.^[^ ^12^ ^]^ Indeed, the presence of IL‐23 or IL‐6 alone is insufficient to induce a sustained IL‐17 expression and stabilization of a pathogenic Th17 phenotype.^[^ ^13^, ^14^ ^]^ Consequently, in our InflammaSkin® model, the in situ activation of skin‐resident Th17 cells is achieved by intradermal injection of IL‐2, anti‐CD3 and anti‐CD28 antibodies and sustained by addition of IL‐1β, IL‐23 and TGF‐β to the culture medium. Using this strategy, various T‐cell subsets (both CD4^+^ and CD8^+^ T cells and γδ T cells) will be activated into IL‐17‐secreting T cells. Furthermore, although IL‐2 is an autocrine factor expressed by T cells upon activation,^[^ ^15^ ^]^ injection of IL‐2 was added to ensure an immediate availability of IL‐2 to activated T cells, since lack of adequate amounts of IL‐2 can induce complete arrest of T‐cell activity.^[^ ^16^ ^]^ As described above, IL‐6 is an essential factor for Th17 differentiation. ^[14]^ IL‐6 is not added in our Th17 polarization cocktail since IL‐6 is endogenously induced in the skin due to tissue trauma caused by the process of skin biopsy punching (data not shown).

In the InflammaSkin® model, secretion of the Th17 cytokines IL‐17A, IL‐17AF, IL‐17F and IL‐22 by skin‐resident T cells was achieved after in situ activation and polarization. While we observed secretion of Th17‐associated cytokines in skin models upon in situ T‐cell activation alone, our findings show significantly higher levels of such cytokines in the presence of the Th17 polarizing cocktail. These results suggest that the presence of a Th17 polarizing cocktail can help to sustain the in situ activation of Th17 cells, possibly by preventing environmental skewing of the Th17 phenotype and/or by promoting the capacity of other T‐cell subsets to produce Th17‐associated cytokines. Secretion of the Th1 cytokine IFN‐γ was also observed, indicating that activation of Th1 cells is occurring following in situ activation in the presence of a Th17 polarization cocktail. However, the presence of Th1 cells is also observed in psoriasis skin and therefore the InflammaSkin® model recapitulates psoriasis features not limited to only Th17‐driven inflammation.

Other skin models using in situ T‐cell activation and Th17 polarization have been recently published and share some similarities to the InflammaSkin® model.^[^ ^17^, ^18^ ^]^ The model described by Smith SH et al^17^ employs skin samples defatted and dermatomed to 750 µm, which could imply that some cellular architecture and dermal immune cells may not been fully represented. Importantly, the Th17 polarization cocktail used in this model does not contain IL‐23, which is reported to be essential for a sustained Th17 differentiation.^[^ ^14^ ^]^ Moreover, the use of animal‐derived substances, such as 64% bovine collagen solution and 2% fetal bovine serum added to the tissue culture wells and to the culture medium, respectively, creates potential variability in the model described by Smith SH et al^[^ ^17^ ^]^ compared with the model described by Garret SM et al^[^ ^18^ ^]^ and to the InflammaSkin® model, which are both developed in 100% serum‐ and animal‐free settings. The model described by Garret SM et al^[^ ^18^ ^]^ is based on an ex *vivo* culture of defatted but not dermatomed skin from healthy individuals, stimulated with different differentiation cocktails, but all containing anti‐CD3 and anti‐CD28 antibodies, IL‐1β and IL‐23 and thus has more similarity to the InflammaSkin® model. However, in their model, skin biopsies are cultured in an air‐liquid interphase where the dermis is submerged directly in the culture medium, creating an artificial wet setting for the dermal compartment that may induce spongiosis due to continuous absorption of liquid.^[^ ^18^ ^]^ Consequently, the feasibility to culture skin tissue in this condition is limited to a short period (48 hours), as described by Garret SM et al^[^ ^18^ ^]^ Furthermore, culture conditions used in their setting (3‐5 biopsies of 3 mm diameter per well in a semi‐submerged condition) does not allow topical application of formulations, limiting the use of this model to test therapeutic strategies. On the contrary, the InflammaSkin® model is based on the NativeSkin® explant technology, by which the skin biopsies are embedded in a proprietary biological matrix inside trans‐well inserts that are placed in wells containing proprietary culture medium. In this system, the epidermal compartment of skin biopsies is left in contact with the air, while the dermal compartment is embedded in a moist, but not wet, environment with constant nutrients supplied by the adjacent culture media and thus resembling more closely a physiological cutaneous environment enabling maintenance of skin viability and integrity.^[^ ^11^ ^]^ Due to the NativeSkin® explant technology, the skin explant models, including InflammaSkin®, can be cultured for at least 7 days. Moreover, the presence of a silicon ring at the surface of the skin biopsy enables repeated topical applications of compounds in a reproducible manner by delimiting a defined treatment area and preventing any lateral diffusion.

We further characterized the InflammaSkin® model for its capacity to establish an interplay between activated Th17 cells and keratinocytes, as exemplified by the increased expression of the epidermal markers, Keratin‐16 and S100A7, known to be highly upregulated in psoriasis lesions.^[^ ^19^ ^]^ Importantly, we provide evidence that the psoriasis‐like inflammatory interplay between Th17 cells and keratinocytes can be inhibited by topical treatment with two classes of drugs of therapeutic relevance for psoriasis, a corticosteroid and a PDE4 inhibitor. Treatment effect was observed for both prophylactic and therapeutic treatment regimes; however, less effect was seen in the therapeutic setting. Importantly, pharmacological studies employing systemic treatments delivered either in the culture media or by subcutaneous injection can also be performed, the latter using a variant of the InflammaSkin® model containing a defined thickness of adipose tissue to support reproducible injection of biologics without any leakage.^[^ ^20^ ^]^

In conclusion, here we describe the InflammaSkin® model, a novel fully human Th17‐driven skin inflammation model, based on in situ activation of skin‐resident Th17 cells that promote psoriasis‐like inflammatory responses in human skin. We provide evidence that (a) the InflammaSkin® model reliably reproduces the cellular interplay between Th17 cells and keratinocytes and features of skin inflammation as observed in psoriasis lesions and (b) the InflammaSkin® model can be used as a preclinical model system to assess efficacy of test compounds with different drug modalities and administration.

## CONFLICT OF INTEREST

The authors declare that there is no conflict of interest. CJ, AD, EB and PD are employed by Genoskin. JH, HN and PL are employed by LEO Pharma A/S. Genoskin and LEO Pharma A/S co‐funded this project.

## **AUTHOR** **CONTRIBUTIONS**

CJ, PD, HN and PL conceived and designed the study. CJ, AD, EB, JLG, JH, HN and PL were involved in acquisition, analysis and interpretation of data. CL, HN and PL wrote the manuscript. All authors have read and approved the final manuscript.

## Supporting information


**Figure S1.** Evaluation of time course for induction of inflammation.

**Figure S2.** Characterization of cytokine expression consecutive to induction of Th17/Th1 inflammation.

**Figure S3.** Characterization of histological changes in epidermal differentiation markers expression consecutive to induction of Th17/Th1 inflammation.

**Figure S4.** Pharmacological response of InflammaSkin® model to therapeutic treatment with BDP gel and PDE4 inhibitor cream.

**Figure S5.** Pharmacological response of InflammaSkin® model to therapeutic treatment with BDP gel and PDE4 inhibitor cream.

**Figure S6.** Pharmacological response of InflammaSkin® model to therapeutic treatment with BDP gel and PDE4 inhibitor cream.
