# Supplementary figures and images for: Development and characterization of a human Th17‐driven ex vivo skin inflammation model
Source: Exp Dermatol. 2020 Aug 25;29(10):993–1003. doi: 10.1111/exd.14160 (PMC7693225; doi:10.1111/exd.14160)

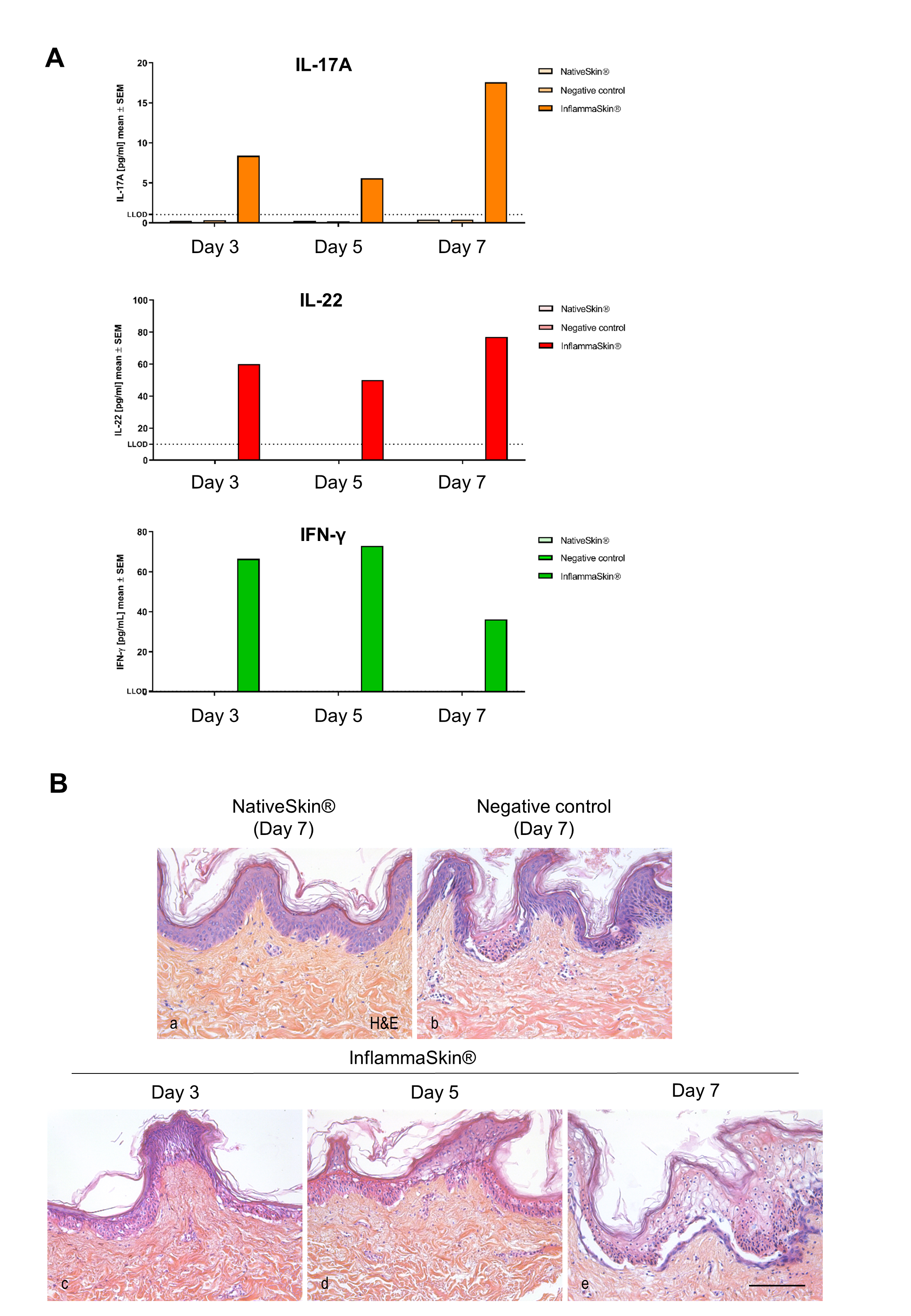

Supplement: Supplementary file 1 — Figure S1. Evaluation of time course for induction of inflammation. [file EXD-29-993-s001.png]

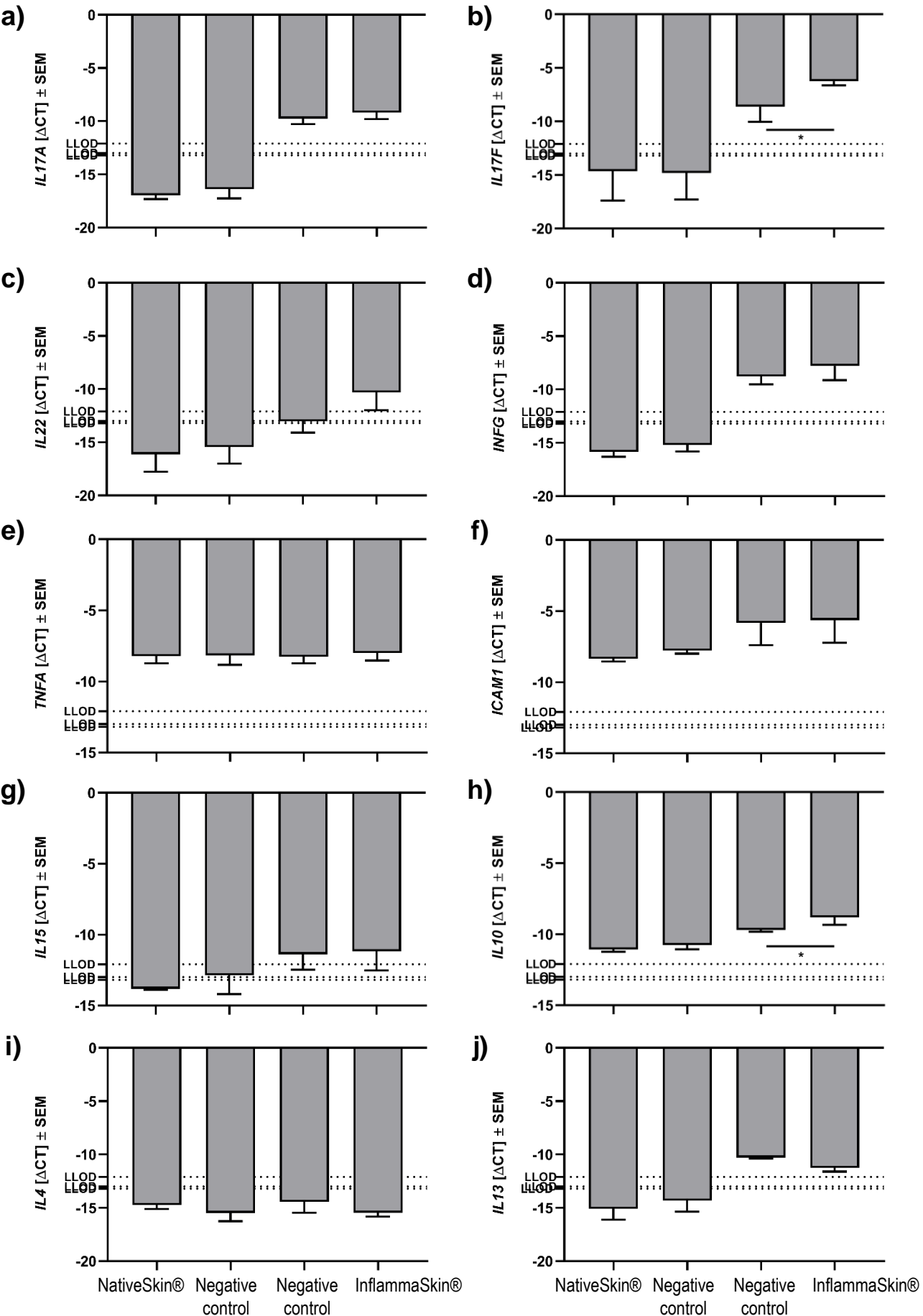

|                           |   |   |   |   |  |   |   |   |   |  |
|---------------------------|---|---|---|---|--|---|---|---|---|--|
| Polarizing Th17 cytokines | - | + | - | + |  | - | + | - | + |  |
| In situ T cell activation | - | - | + | + |  | - | - | + | + |  |

Supplement: Supplementary file 2 — Figure S2. Characterization of cytokine expression consecutive to induction of Th17/Th1 inflammation. [file EXD-29-993-s002.PDF]

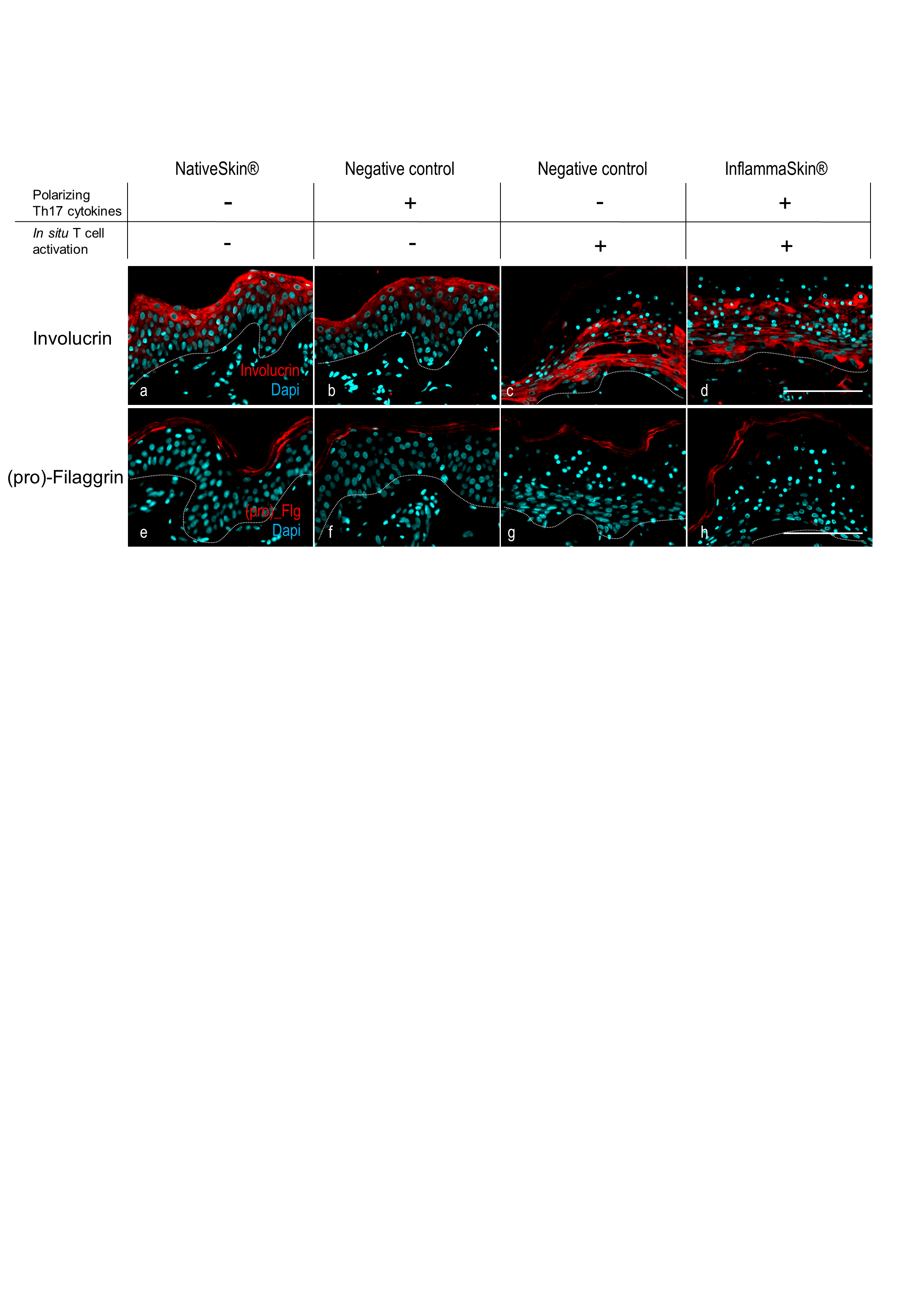

Supplement: Supplementary file 3 — Figure S3. Characterization of histological changes in epidermal differentiation markers expression consecutive to induction of Th17/Th1 inflammation. [file EXD-29-993-s003.PNG]

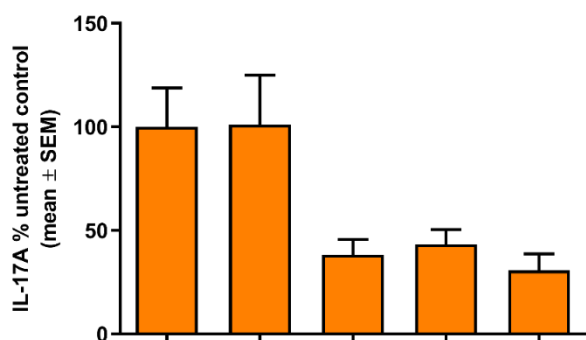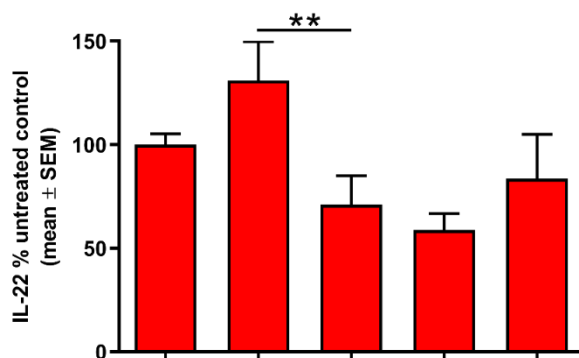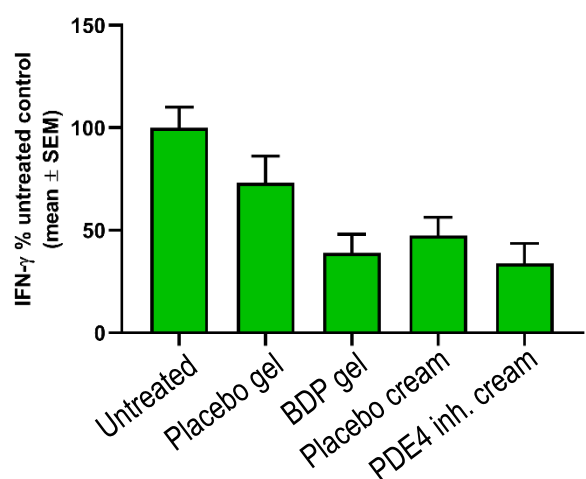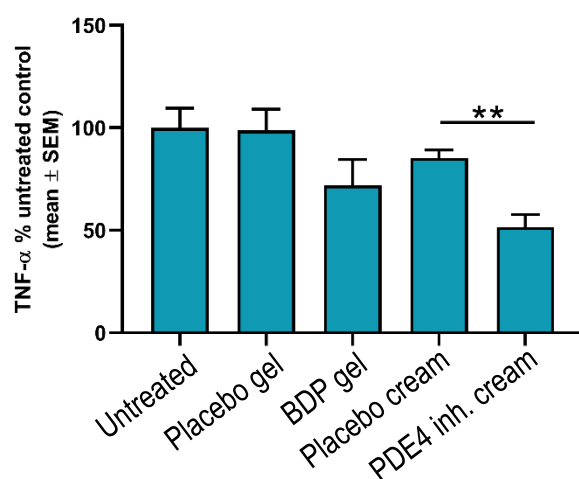

Supplement: Supplementary file 4 — Figure S4. Pharmacological response of InflammaSkin® model to therapeutic treatment with BDP gel and PDE4 inhibitor cream. [file EXD-29-993-s004.pdf]

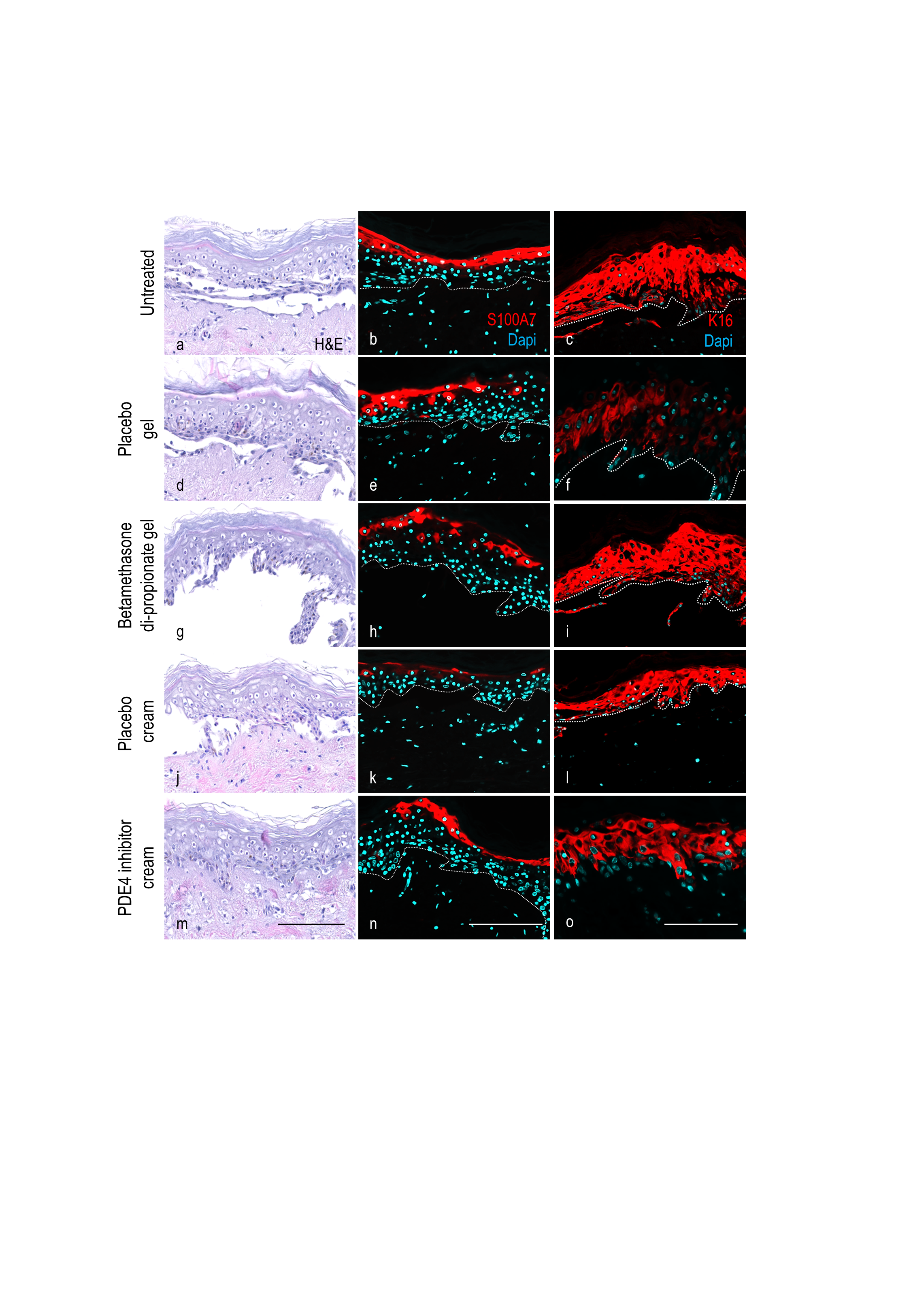

Supplement: Supplementary file 5 — Figure S5. Pharmacological response of InflammaSkin® model to therapeutic treatment with BDP gel and PDE4 inhibitor cream. [file EXD-29-993-s005.png]

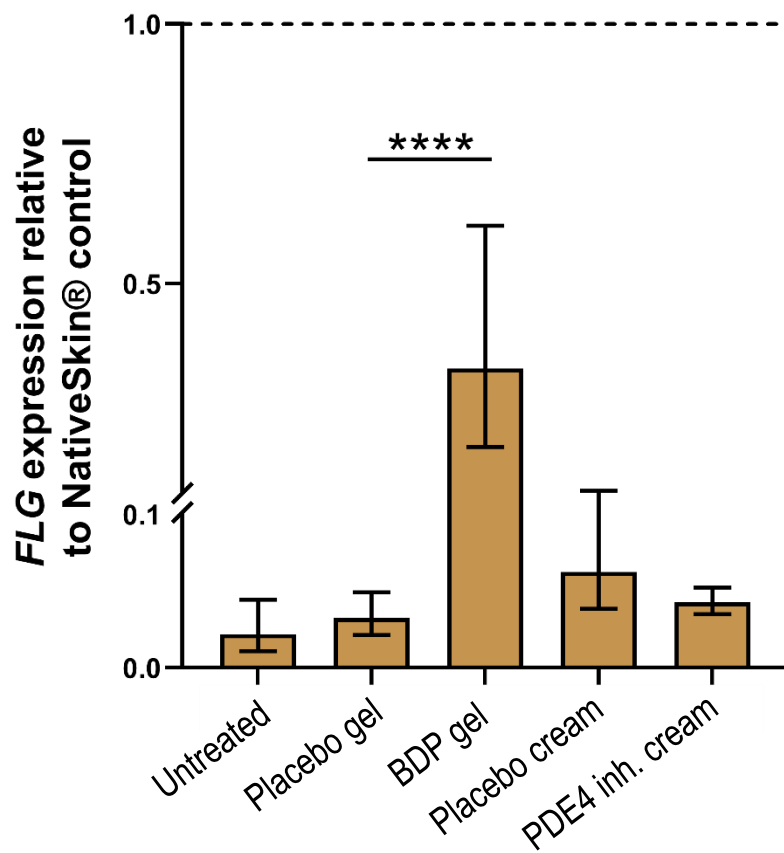

Supplement: Supplementary file 6 — Figure S6. Pharmacological response of InflammaSkin® model to therapeutic treatment with BDP gel and PDE4 inhibitor cream. [file EXD-29-993-s006.pdf]
